# Supplementary material for: Glutathione contributes to efficient post-Golgi trafficking of incoming HPV16 genome
Source: PLoS One. 2019 Nov 19;14(11):e0225496. doi: 10.1371/journal.pone.0225496 (PMC6863556; doi:10.1371/journal.pone.0225496)
Supplement: Attachment — Submitted filename: response letter.docx [file pone.0225496.s001.docx]

**Response to Reviewers (responses in red)**

Reviewer #1: Cellular entry of papillomaviruses is a complex process that is only partly understood. A critical stage of entry involves the penetration of a portion of L2 across the endosomal membrane to facilitate trafficking of vesicles containing the viral genome to the nucleus. In this paper, Li et al show that inhibition of the cellular glutathione system through drug treatment of knockdown of GSH-generating enzymes results in less efficient penetration of L2 into the cytoplasm, trafficking of the virus to the nucleus, and consequently virus infection. This is a nice paper, well executed and well presented, and the data are convincing. A few points/questions should be addressed:

1. The authors make the point (lines 326-330) that BSO treatment does not affect the cell cycle. They say that there was “a subtle but statistically insignificant expansion of S phase upon BSO treatment.” If the expansion is not statistically significant, then they cannot say that there is an expansion. It also does not help their case to claim that there is one. Better to say something like “no statistically significant changes in cell cycle profiles were observed”, and leave it at that.

Thank you for raising this concern but we feel that these subtle effects of BSO may indeed be biologically relevant, even if they are not statistically “significant” based on the commonly used p-value threshold of 0.05. We feel it’s better to be upfront in reporting these differences and say they were not significant rather than to ignore or dismiss them, especially because these subtle trends were seen across multiple independent experiments. The text has been slightly modified to better reflect this perspective.

2. In figure 3C, there seems to be an increase in the levels of the 25kD degraded L1 band in BSO vs control. Was this consistently observed? The authors should quantify their western results across several blots.

Thank you for pointing this out. Further analysis of multiple replicates do indeed reveal a significant difference in degraded L1 at this 2h time point. We have performed densitometry of these reps and include that analysis as new panels in figure 3. Text has been modified accordingly.

3. The graph in Fig 4D is kind of hard to read with the little symbols. It would be better to indicate the things being quantified with labels under the X axis.

We have modified figure 4, and directly labeled the X-axis as suggested.

4. In the discussion, the authors discuss ways that GSH might affect vesicular trafficking. This may important, but their own data in Fig 5 show that penetration of L2 C terminus into the cytoplasm is the key step. Failure to translocate L2 would be sufficient to explain the failure in trafficking of HPV-containing vesicles. In that light, the authors should discuss more about what is known about that translocation step. How might cellular redox affect it? What is the relationship between redox in the cytoplasm and redox in the endosomal lumen, where L2 would be present prior to translocation? Does L2 have redox-sensitive cysteines?

Our data indicated failure of L2/vDNA to traffic post-Golgi. This could be due to failure of L2/vDNA to bind mitotic chromosomes or failure of L2/vDNA to egress/traffic from the vesiculating Golgi. We added a bit to what is known about the post-Golgi trafficking and translocation step in the discussion, lines 464-475.

5. Would knockdown of the GSH production machinery affect the levels of NADPH? Is it known what effect high NADPH might have on the cell?

We did not directly look at NAD+/NADH (or NADP/NADPH) levels, but GSH depletion certainly affects these redox couples which may indeed affect many aspects of cellular physiology including DNA damage (via NAD+ dependent PARP activity), survival, and ROS responses. All these probable effects add to the challenges of understanding the molecular basis for the observed phenotype. We chose not to comment or speculate too much in this regard.

Reviewer #2: Overall, this manuscript provides relevant and interesting insight into what has been an experimentally challenging question – that of understanding, at a molecular level, how HPV virions enter the cell and begin their life cycle. While the insight now provided regarding how the virions exit the trans-Golgi is somewhat general, it does provide a useful framework and sets the stage for a more detailed molecular explanation in the future.

Specific comments are noted below.

• A figure showing virion entry and trafficking, highlighting the areas where glutathione/redox is likely to be important, would help the reader to navigate the manuscript.

We have added a final summary figure 6 based on the recommendation.

• Have the authors definitely established that it is the specific concentrations of the actual GSH molecule that are required, or could the requirement be something more general, such as redox potential? It may be possible to provide experimental evidence one way or the other; alternatively, perhaps the language could be broadened somewhat. (For example, see page 20, lines 443-445).

We currently cannot say whether it’s the GSH molecule itself, the GSH/GSSG redox potential, or downstream effects on other redox couples. We have broadened the language in the discussion to reflect this, lines 471-477.

Reviewer #3: This manuscript from the Campos group highlights a key role for aspects of the cellular redox system in HPV16 infection. Using compounds and siRNA depletion of key cellular factors coupled to established (and well controlled) assays they demonstrate that the role of these factors appears to occur in post-Golgi trafficking. The study is of interest to those working out the convoluted infection process of HPV. The data are tight and the manuscript well written. I do not see a need to ask the authors to perform more experiments when the present study is sufficient.
